# Risk assessment of a mixture of pharmaceuticals and personal care products (PPCPs) in thermal waters of Kütahya, Türkiye

**DOI:** 10.1007/s10653-026-03082-2

**Published:** 2026-02-21

**Authors:** Harun Şener, Evşen Yavuz Güzel, Hüseyin Karakuş

**Affiliations:** 1 Department of Forensic Science, Faculty of Engineering and Natural Sciences, Kütahya Health Sciences University, Kütahya, Turkey; 2 Department of Basic Science, Faculty of Fisheries, Çukurova University, Adana, Turkey; 3 Department of Geological Engineering, Kütahya Dumlupınar University, Kütahya, Turkey

**Keywords:** PPCPs, Thermal water, Risk assessment, LC–MS/MS, Groundwater

## Abstract

The increasing global consumption of pharmaceuticals and personal care products (PPCPs) and their persistence in the environment have raised concerns about their potential ecological and human health risks, especially in aquatic systems. Thermal waters, widely used for therapeutic and agricultural purposes, remain underexplored regarding PPCP contamination. This study presents a comprehensive environmental risk assessment (ERA) of 104 PPCPs in thermal waters from 22 sampling points across nine geothermal fields in Kütahya, Türkiye. A total of five PPCPs (caffeine, DEET, ephedrine, carbamazepine, and chlorphenamine) were quantified using a validated LC–MS/MS method. Caffeine exhibited the highest detection frequency (100%) and maximum concentration (43.191 ng L^−1^), followed by DEET (90.9%) and ephedrine (77.2%). Risk quotients (RQs) were calculated based on predicted no-effect concentrations (PNECs) derived from ECOSAR v2.2, and the mixture risk quotient (MRQ) approach was applied to assess the cumulative ecological risks of PPCP mixtures. The results indicate that although most individual compounds pose low to medium ecological risk levels (RQ < 1), MRQ values exceeded concern thresholds (MRQ > 1) in several locations, suggesting potential chronic threats, especially to algae and invertebrates. No significant correlation emerged between PPCP levels and water parameters, indicating their persistence. Geological and hydrogeological factors, such as fault-controlled mixing between shallow and deep aquifers, were identified as pathways for contamination. These findings highlight the need for regulatory oversight of PPCPs in thermal waters and recommend safeguards, such as isolating well intakes from shallow aquifers, to reduce ecological and health risks.

**Supplementary Information:**

The online version contains supplementary material available at 10.1007/s10653-026-03082-2.

## Introduction

The rapid growth of the global human population, coupled with intensified anthropogenic activities such as industrialization, transportation, agriculture, animal husbandry, pharmaceutical manufacturing, and urbanization, has led to a substantial increase in the release of pollutants into the environment (Lukač Reberski et al., 2022; Misstear et al., 2023). Over the past few decades, there has been a marked rise in the production and consumption of pharmaceutical and personal care products (PPCPs), driven by the need to meet modern lifestyle demands and enhance healthcare systems (Silori et al., 2022). PPCPs comprise a broad range of natural and synthetic compounds used in pharmaceutical formulations across various therapeutic classes for both human and veterinary applications, as well as in personal care items such as soaps, lotions, toothpaste, and sunscreens, which are routinely used in large quantities worldwide (Daughton & Ternes, 1999). The pervasive and increasing use of PPCPs has contributed to their widespread detection in diverse environmental matrices across all continents (Hawash et al., 2023; Silori et al., 2022). The active ingredients in pharmaceutical and personal care products are specifically designed to interact with targeted organs or receptors in humans and animals. However, unintended effects may occur in non-target organs or species (Boxall et al., 2012). Consequently, the continuous and long-term introduction of PPCPs into the environment may result in subtle, chronic, or cumulative adverse effects on both aquatic and terrestrial organisms, some of which may only become evident after several decades (Sengar & Vijayanandan, 2022). In addition, long-term persistence and partial degradation in the environment make the accumulation of PPCPs in water bodies a legitimate concern (Silori et al., 2022). Their presence in aquatic environments such as surface water, groundwater, and wastewater has raised concerns for both human health and aquatic life due to their potentially toxic compositions and degradation processes (Silori et al., 2022).

One of the primary pathways through which PPCPs enter aquatic environments and ultimately drinking water sources is via wastewater treatment plants. Several studies have indicated that the contamination of groundwater by PPCPs can occur as a result of irrigating agricultural fields with treated wastewater (Silori et al., 2022). Additional sources include effluents from pharmaceutical industries, hospital wastewater, fecal waste from humans and animals, landfills, livestock operations, and aquaculture activities (Silori & Tauseef, 2022). Owing to the diverse physicochemical properties of PPCPs such as their hydrophilicity, solubility, volatility, and biodegradability, conventional sewage treatment plants, which are burdened with a broad spectrum of chemical compounds from domestic and industrial sources, are often insufficiently effective in removing these contaminants (Silori et al., 2022; Wydro et al., 2024).

Although groundwater is generally less vulnerable to contamination than surface water owing to physical, chemical, and biological attenuation processes occurring in the subsurface, residues of numerous PPCPs have nonetheless been detected in groundwater systems (Lukač Reberski et al., 2022; Mukhopadhyay et al., 2022; Stefano et al., 2022; Yang et al., 2018) as well as in thermal pool waters (Jakab et al., 2020), with concentrations ranging from picograms per liter (pg/L) to nanograms per liter (ng/L). The frequent detection of PPCPs in drinking water, wastewater treatment plants (WWTPs), and groundwater is attributed to their widespread use, improper disposal by manufacturers, limited metabolic breakdown in humans, and persistent biological activity (Hawash et al., 2023). Despite the potential ecological and human health risks, knowledge regarding the toxicity, environmental behavior, and long-term effects of many PPCPs remains limited. As a result, only a small number are routinely monitored, and the majority remain unregulated (Sui et al., 2015). In the EU, binding groundwater quality standards are set at Union level for nitrates and pesticides, while additional pollutants are addressed through Member State threshold values; the 2014/80/EU amendment also highlights the need for a groundwater watch list including emerging pollutants (European Commission  2006, 2014). In this context, comprehensive investigations into the occurrence and behavior of unregulated CECs in urban groundwater are essential to address this regulatory gap (Labad et al., 2023). However, compared with surface waters and conventional shallow groundwater, evidence on PPCP occurrence in thermal waters is still scarce and largely limited to a small number of case-specific investigations, and the controlling hydrogeological pathways and mixture-related ecological risks remain poorly constrained, even though these waters are widely used for balneological and recreational purposes and may be vulnerable to anthropogenic inputs through mixing with shallow flow paths (Jakab et al., 2020; Silori et al., 2022).

As with surface waters, no acceptable concentration thresholds for PPCPs have been established in thermal waters for environmental risk assessment. Existing environmental risk assessment frameworks for PPCPs recommend the use of predicted no effect concentrations (PNECs), which are derived by identifying the most sensitive endpoints from ecotoxicological tests conducted across three trophic levels (algae, crustaceans, and fish) and applying an appropriate assessment factor (AF) (EMA, 2006). More recent research has shifted toward the development of novel approaches that assess the cumulative effects of chemical mixtures in the environment, rather than focusing solely on the impact of individual compounds (Backhaus & Faust, 2012; Reis et al., 2021; Riva et al., 2019). Given the distinctive physicochemical properties of thermal waters, the complexity of hydrogeological settings, and the potential for anthropogenic influence through balneological practices, thermal systems represent a critical yet understudied component of aquatic environments with respect to PPCP contamination (Jakab et al., 2020; Silori et al., 2022). Furthermore, current regulatory frameworks rarely incorporate thermal water systems within their monitoring and assessment strategies for emerging contaminants. Thermal waters are often used in open systems that involve direct and repeated human contact, and large volumes of spent water may be discharged into the surrounding environment with limited treatment. Therefore, baseline occurrence data and mixture-oriented ecological risk screening for thermal waters, particularly in geothermal provinces with intensive usage, such as Kütahya, remain insufficient to support evidence-based monitoring and protection strategies.

In light of these considerations, this study was designed to investigate the occurrence, spatial distribution, and environmental risk characteristics of selected PPCPs in thermal waters sampled from diverse geothermal settings across the Kütahya region in western Türkiye. The study aimed to evaluate both individual compound risks and the cumulative effects of PPCP mixtures by applying established environmental risk assessment methodologies based on ecotoxicological data. Through this approach, the present study provides a scientific basis for understanding the vulnerability of geothermal systems to emerging contaminants and supports the development of tailored protection strategies for sustainable groundwater management in Türkiye and similar hydrothermal regions.

Accordingly, this study aimed to screen for 104 targeted PPCPs and quantify detected compounds in thermal waters collected from 22 outlets across nine geothermal fields in Kütahya, Türkiye, using a validated LC–MS/MS method. In addition, we conducted a screening-level ecological risk assessment for individual PPCPs and their mixtures using PNEC-based RQ and MRQ metrics, and interpreted spatial variability and plausible contamination pathways in the context of the regional hydrogeological setting and measured physicochemical parameters.

## Materials and methods

### Study area

**Fig. 1:**
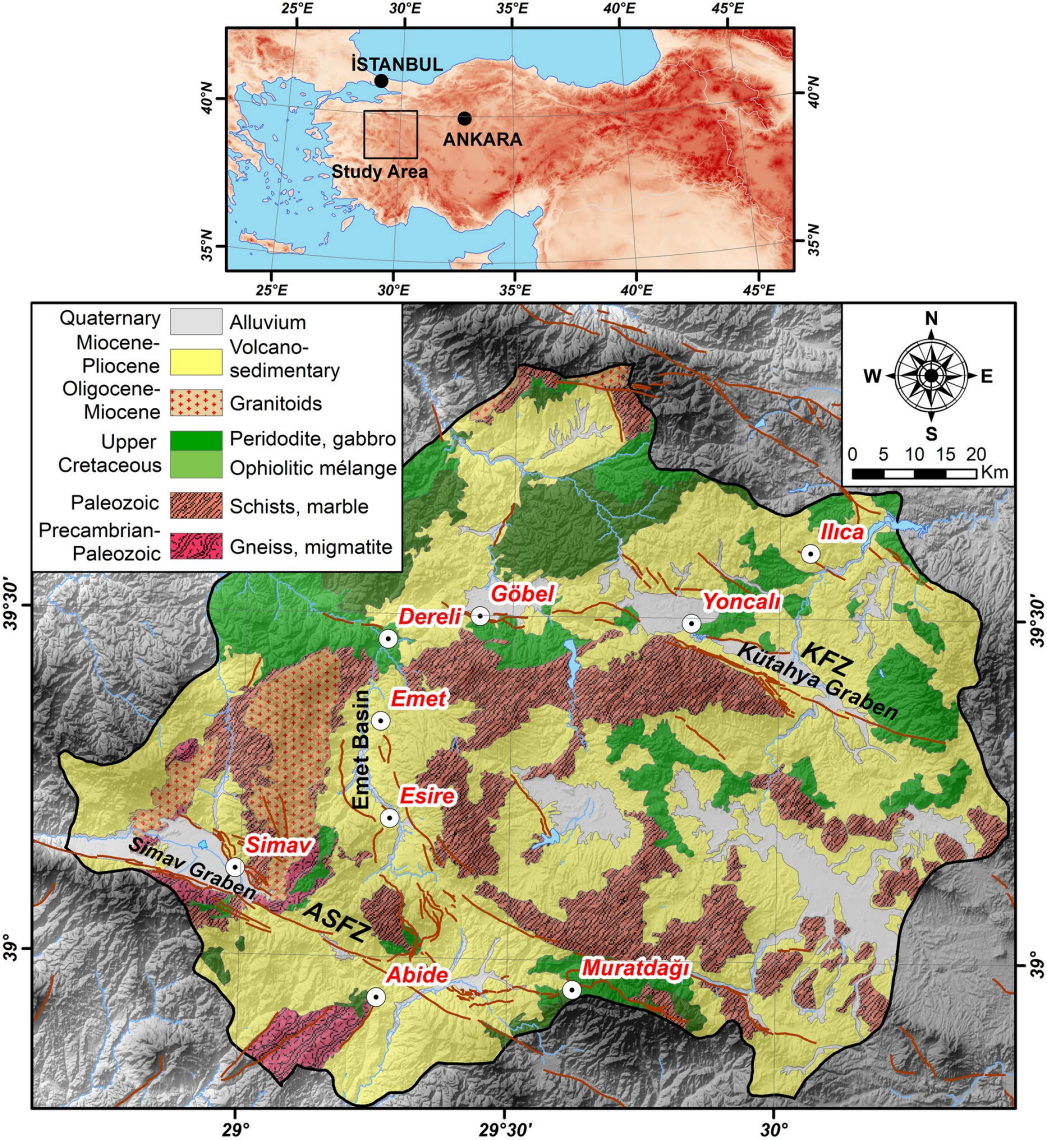
Generalized geological map of Kütahya province. Dark brown lines show active faults from (Emre et al., 2018). ASFZ: Akşehir-Simav Fault Zone, KFZ: Kütahya Fault Zone

Kütahya Province (western Türkiye) hosts several geothermal systems within a fault-controlled extensional tectonic regime characterized by horst-graben structures. Active fault zones act as conduits for geothermal upflow and may promote local mixing between deep thermal fluids and shallow groundwater, which is pertinent for the sampling design and for interpreting spatial differences among sites. Geothermal fields are distributed along the main tectonic domains (Akşehir-Simav Fault Zone, Kütahya Fault Zone, and the Emet Basin), where active faults act as conduits for geothermal upflow and may facilitate local mixing between deep thermal fluids and shallow groundwater, which is relevant for interpreting spatial differences among sampling sites (Koçyiğit & Deveci, 2005). The sampling locations are shown in Fig. 1, and site-specific information is provided in Table S1.

### Sample collection

In January 2021, 3 L of water samples were collected from each of the 22 sampling point, which comprised 9 natural springs (depth = 0 m), 8 pumped wells (72–500 m), and 5 artesian wells (6–602 m). Discharge temperatures ranged from 33 to 152 °C and flow rates from 1.1 to 73 L s^−1^. Site locations are reported as UTM coordinates (ED50, Zone 35), and full site-specific metadata (coordinates, outlet type, depth, temperature, and discharge) are provided in Table S1. Prior to sample collection, the glass bottles were first rinsed with acetone, followed by ultrapure water. Each bottle was then washed twice with water from the sampling points before the final sample collection. The bottles were sealed with glass stoppers to prevent contamination. Samples were directly collected from the source points of the Hisarcık/Esire, Gediz/Murat Dağı, Kütahya/Ilıca, Tavşanlı/Göbel, and Emet springs, where thermal water naturally emerges at the surface. Thermal waters at the Gediz/Ilıca and Simav locations are discharged from artesian wells with high temperature and pressure. Samples from these locations were collected using glass bottles and aluminum funnels attached to the wellhead. Samples from the Kütahya/Yoncalı region, where thermal water is extracted using downhole pumps, were collected through HDPE (high-density polyethylene) pipes.

### Chemicals and standards

In this study, all reference standard materials, including PPCPs and internal standard (IS), were purchased from Dr. Ehrenstorfer in Augsburg, Germany, ensuring that they met certified purity levels of above 98%. For the experiments, we obtained LC-grade methanol (MeOH) and ultrapure water from Merck, both with purity levels ranging from 99.8% to 100%, sourced from Darmstadt, Germany. An OASIS HLB cartridge (3 cc, 60 mg) for solid phase extraction (SPE) was acquired from Waters Corporation in Milford, MA, USA. Each day, we prepared working solutions by diluting the mixed stock standards along with an IS solution, both of which were held at a concentration of 10 ng mL^−1^ in methanol. To prepare quality control (QC) samples and calibrators, we performed a series of dilutions of the mixed working solution in a blank water sample. We consistently maintained an IS concentration of diazepam-d5 and triclosan-d3, resulting in individual analyte concentrations ranging from 0.1 to 100 ng mL^−1^. All stock and working solutions were securely stored at − 20 °C in dark glass bottles to maintain their integrity.

### Sample preparation and extraction

Sample preparation and SPE were performed according to U.S. EPA Method 1694 with minor modifications. Briefly, 1 L of each thermal water sample was filtered through a GFC glass-fibre filter and acidified to pH 2 using sulfuric acid prior to SPE and LC–MS/MS analysis (USEPA 2007). Subsequently, 10 µL of a 1 ppm diazepam-d5 and triclosan-d3 solution was incorporated into the samples, serving as an internal standard for the subsequent analysis. The filtered thermal water samples were then introduced into conditioned OASIS HLB cartridges (3 cc, 60 mg), which had been pre-conditioned with 5 mL of methanol followed by 5 mL of water at a pH of 2, executed at a flow rate of 10 mL.min^−1^. After the loading process, the cartridges were dried under a nitrogen atmosphere for 30 min. Finally, the samples were eluted with 10 mL of methanol containing 6% ammonia, dried completely under nitrogen, re-dissolved in 1 mL of methanol, transferred to vials, and subsequently prepared for LC–MS/MS analysis.

### Instrumental analysis

The investigation into 104 PPCPs present in thermal water samples was carried out utilising a Shimadzu 8040 LC–MS/MS system based in Kyoto, Japan. The chromatographic separation of the analyses was performed at room temperature on a Shim-pack column (150 × 2.0 mm ID) manufactured by Shimadzu Corporation, also located in Kyoto, Japan, with a constant flow rate of 0.4 mL/min. The separation of PPCPs was facilitated through an oven program, employing mobile phases consisting of 5 mM ammonium formate in water (designated as A) and methanol (referred to as B). The chromatographic gradient was initiated in positive ionization mode with a mobile phase composition of 10% solvent B, maintained for 10 min at a flow rate of 0.4 mL min^−1^. Subsequently, the flow rate was increased to 1.0 mL min^−1^ with a corresponding increase in solvent B to 90% for a duration of 5 min. Thereafter, the system returned to the initial conditions of 10% solvent B and a flow rate of 0.4 mL min^−1^ over the following 15 min. During the first 1.5 min, the eluent was directed to waste, culminating in a total run time of 18 min. The analytes were scrutinized in the positive ionization mode (ESI +), with their identification and quantification conducted via two distinct transitions (MRM mode). Retention times (in minutes), precursor ions (m/z), and product ions (m/z) for a comprehensive total of 104 target analytes and their ISs are detailed in Table S2 of the Supplementary File. Analytical method validation was completed according to the validation parameters of carryover, interferences, limit of detection, limit of quantitation, matrix effects, processed sample stability, extraction efficiency (recovery), and reproducibility with guidance from the ANSI/ASB Std. 036 (AAFS Standards Board, 2019) method validation guidelines. To exclude laboratory contamination, solvent and procedural blanks were analyzed throughout each LC–MS/MS sequence and showed no target PPCPs above the method detection limits. QC samples and calibrators were prepared by spiking blank pure water with methanolic working solutions prepared from reference standards. Diazepam-d5 and triclosan-d3 was used as the internal standard (IS) for quantification. The calibration curve runs consisted of eight calibrator samples that ranged from 0.02 to 250 ng L^−1^ for all analytes. All real samples, QC samples, neat standards, and calibrators were analysed on LC–MS/MS after the above extraction procedure, and then validation results were characterised according to the peak areas, nominal and measured concentrations obtained.

### Statistical analysis

The frequency of positive detection (freq), expressed as a percentage, along with the arithmetic mean, median (med), and 90th percentile (Per90), was calculated using Microsoft Excel 2016 (Microsoft Corporation, USA). In these analyses, non-detected values were assigned a value of zero.

## Environmental risk

### ERA for single PPCPs

The environmental risk of PPCPs was assessed using the Risk Quotient (RQi) approach. This method enables the assessment of the risk posed to the most sensitive trophic level by each PPCP (Guzel et al., 2018; Molnar et al., 2021). RQi is calculated as the ratio of the measured environmental concentration (MECi) to the PNECi in thermal waters (Eq. 1). PNECi was obtained as the ratio between the concentration of substance i that represents 50% of its maximal effect in short-term tests (Effect concentration—EC50) and the Assessment Factor (AF) (AF: 1000), which takes into account analytical and biological variability (Backhaus & Karlsson, 2014; Bouissou-Schurtz et al., 2014; Riva et al., 2019). The magnitude of the AF can range from 10 to 1000 and depends on the extent of knowledge about the system: the less is known, the larger the factor should be (Nikinmaa, 2014).

1 $$RQi=\frac{MECi}{PNECi}$$

To represent a worst-case scenario, the environmental risk assessment was conducted using the highest detected concentrations of each PPCP in thermal waters.

Table S3 presents the (PNECs) for the selected PPCPs. These values were calculated using EC50 and/or LC50 data obtained from the ECOSAR v2.2 program (see Table S3). Risk Quotient (RQ) values are commonly categorized into three risk levels: RQ > 1 indicates high risk, 0.1 < RQ ≤ 1 indicates medium risk, and 0.01 < RQ ≤ 0.1 indicates low risk (Hernando et al., 2006).

### ERA for a mixture of PPCPs

Assessing the environmental risk of PPCP mixtures is essential for a more comprehensive evaluation of ecological impacts, particularly when individual compounds occur at low-risk or non-risk concentrations. To address this, Backhaus et al. proposed the use of the concentration addition (CA) concept, which enables the estimation of cumulative risk through a stepwise approach (Backhaus & Faust, 2012; Backhaus & Karlsson, 2014). In this study, the mixture risk quotients (MRQs) were calculated using two different methods, as outlined in Eq. 2, to evaluate the overall risk posed by the combined presence of PPCPs.

2 $$ {\mathrm{MRQ}}_{{{\mathrm{MEC}}/{\mathrm{PNEC}}}} = \mathop \sum \limits_{i = 1}^{n} \frac{MECi}{{PNECi}} $$

Here, the MRQ represents the sum of the highest individual RQ values for the detected PPCPs.

## Results and discussion

LC–MS/MS analysis was employed to determine the occurrence of PPCPs in source thermal water discharges collected at springs and wellheads. These thermal waters are situated in urban, agricultural, and rural areas and are primarily used for balneological and thermal tourism purposes. However, in the Simav region, thermal waters are also utilized for greenhouse and district heating systems. A total of five PPCPs were detected in the 22 thermal water samples (Table 1).

**Table 1:** Concentrations (ng L^−1^) and number of detections (n) of PPCPs in thermal water samples from all sampling sites

Compound	Acronym	Therapeutic use	n	min (ng L^−1^)	max (ng L^−1^)
Caffeine	CF	Stimulant	22	6.013	43.191
N,N-diethyl-metatoluamide	DEET	Insect repellent	20	9.892	22.756
Ephedrine	EPH	Stimulant	17	3.793	9.223
Carbamazepine	CBZ	Antiepileptic	5	0.038	0.07
Chlorphenamine	CPM	Antihistamine	5	0.05	0.127

### Occurrence of PPCPs in thermal water

**Fig. 2:**
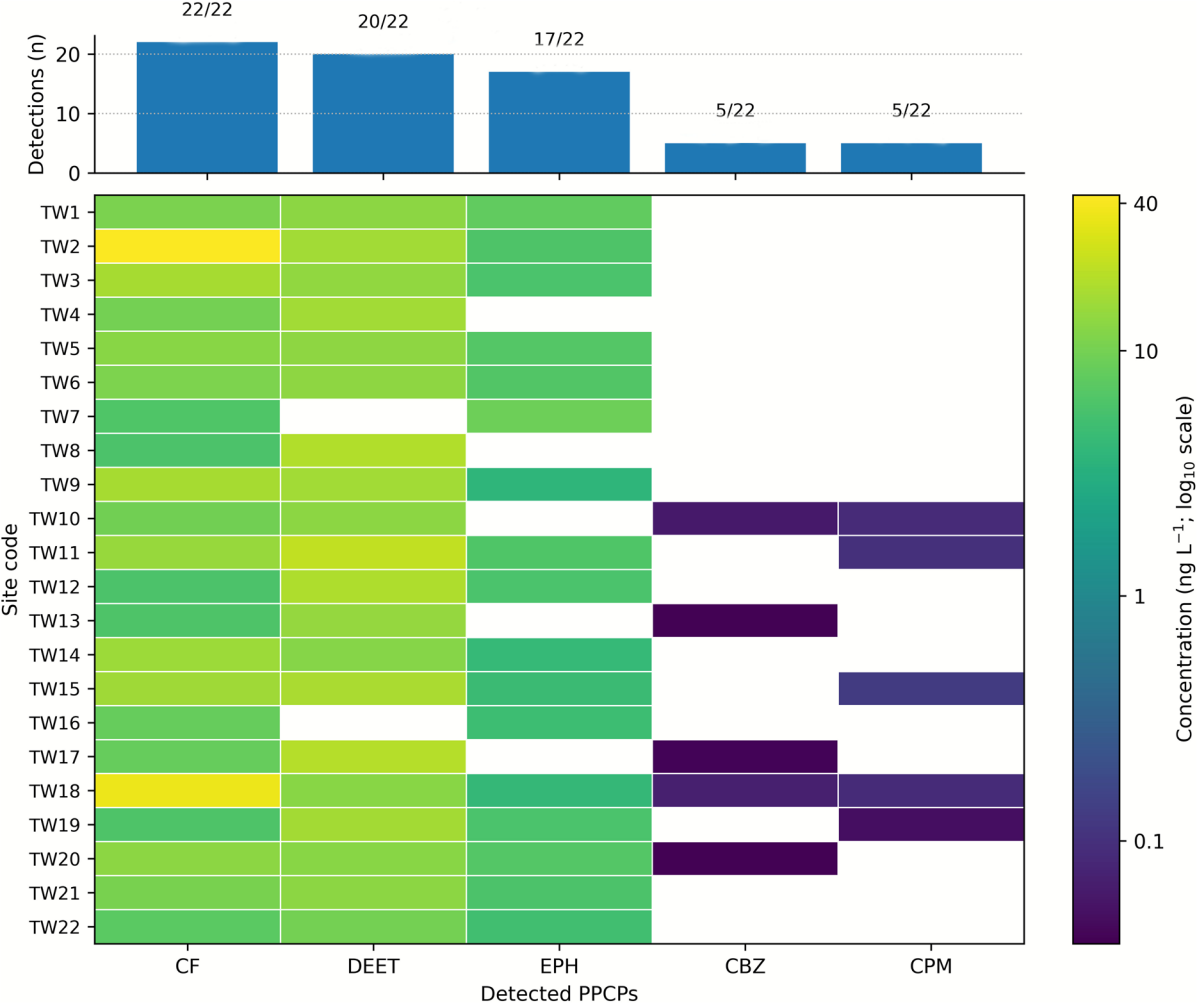
Detection frequency (n) of the quantified PPCPs across 22 thermal water samples. log_10_ concentrations (ng L^−1^) of detected PPCPs (TW: Thermal water)

A total of 104 pharmaceutical compounds from various therapeutic classes, including psychiatric drugs, β-blockers, nonsteroidal anti-inflammatory drugs (NSAIDs), stimulants, and others, were investigated. Five PPCPs were identified and quantified from a list of target analytes, which were selected based on the most commonly prescribed pharmaceuticals in Turkey. The concentrations of the detected compounds were found to be in the low nanogram per liter (ng/L) range. Caffeine was the most frequently detected compound, with a detection frequency of 100%, followed by DEET and ephedrine, detected in 90.9% and 77.2% of samples, respectively. In contrast, carbamazepine and chlorphenamine exhibited the lowest detection frequencies, each present in only 22.7% of the samples. The detection frequencies and site-wise concentration patterns of the quantified PPCPs are summarized in Fig. 2.

Overall, detecting only five PPCPs indicates a narrower contaminant spectrum than typically reported for shallow groundwater strongly influenced by wastewater recharge or riverbank infiltration, where a broader variety of lifestyle chemicals is frequently observed (Loos et al., 2010; Ma et al., 2022; Richards et al., 2021; Sorensen et al., 2015). The generally low ng L^−1^ concentrations in the present thermal waters likely reflect a highly diluted anthropogenic signal and restricted hydraulic communication between the deeper geothermal reservoir and shallow anthropogenically impacted waters. Nevertheless, the ubiquitous presence of caffeine and the high detection frequency of DEET demonstrate that measurable anthropogenic inputs can still reach thermal systems, consistent with concerns raised for balneological waters and thermal facilities as potential sources and receivers of emerging contaminants (Jakab et al., 2020). The five detected compounds (caffeine, DEET, ephedrine, carbamazepine, and chlorphenamine) are among the PPCPs most frequently reported in wastewater-impacted surface waters and groundwater worldwide and are widely used as indicators of anthropogenic pressure (Hawash et al., 2023; Loos et al., 2010; Sui et al., 2015). Accordingly, compared with wastewater-influenced shallow groundwater and riverbank-filtration settings, the concentration ranges measured in the thermal waters fall toward the low end of reported environmental levels (Loos et al., 2010; Ma et al., 2022; Richards et al., 2021; Sorensen et al., 2015).

Carbamazepine, an antiepileptic and mood-stabilizing pharmaceutical with widespread clinical use, is frequently regarded as a conservative indicator of domestic wastewater influence in aquatic environments. Its prominence in groundwater is largely linked to its limited removal during conventional wastewater treatment (Bueno et al., 2012) and resistance to photochemical and biological transformation (Almeida et al., 2021), which together promote persistence during recharge and subsurface transport (Lukač Reberski et al., 2022; Richards et al., 2021). Carbamazepine is hydrolytically stable and is predominantly neutral at environmentally relevant pH, which can limit electrostatic interactions with typically negatively charged aquifer minerals and thereby favor mobility and long-range transport in groundwater (Lukač Reberski et al., 2022). Large monitoring efforts consistently report carbamazepine among the most frequently detected pharmaceuticals across surface-water and groundwater systems, supporting its role as a robust marker of anthropogenic pressure (Loos et al., 2010; Wilkinson et al., 2022). In this study, carbamazepine was detected in a limited subset of samples (22.7%) at trace levels (0.038–0.07 ng L^−1^), which are markedly lower than concentrations commonly reported for shallow groundwater impacted by wastewater-derived recharge. Although the present dataset does not enable definitive source apportionment, the low detection frequency and concentrations suggest that wastewater inputs to the thermal waters are limited and/or that hydraulic connectivity between anthropogenically influenced shallow groundwater and the deeper thermal reservoir is restricted. Nevertheless, the detection of carbamazepine at some outlets indicates that localized anthropogenic signals can reach certain thermal water points.

Caffeine is a widely consumed stimulant present in beverages and some pharmaceuticals and enters municipal wastewater primarily through human excretion and household discharges (Buerge et al., 2003; Gilbert et al., 1976). Due to ubiquitous use and the scarcity of significant natural sources in most freshwater settings, caffeine is often used as a marker of anthropogenic activity and wastewater influence (Buerge et al., 2003; Gonçalves et al., 2017; Hillebrand et al., 2012; Machado et al., 2016; Tran et al., 2014). Although caffeine is generally more biodegradable than many persistent PPCPs, population-scale consumption can generate sufficient loads that residual concentrations remain detectable in receiving waters even when treatment performance is relatively high (Li et al., 2020; Machado et al., 2016). In groundwater, caffeine may attenuate during subsurface transport; therefore, its presence is frequently interpreted as evidence of shorter travel times and relatively recent wastewater contributions rather than long-residence-time contamination (Hillebrand et al., 2012). Consistent with this framework, a pan-European groundwater survey reported caffeine with a detection frequency of 82.9% and associated it with groundwater infiltration and contamination (Loos et al., 2010). In this study, caffeine was detected in all samples at 6.013–43.191 ng L^−1^, supporting caffeine as a sensitive indicator of recent, low-intensity anthropogenic inputs to the sampled thermal systems.

DEET emerged as one of the most consistently observed compounds in the thermal waters. Concentrations ranged from 9.892 to 22.756 ng L^−1^, supporting comparison with better-characterized surface-water and groundwater compartments. The observed range broadly overlaps with concentrations reported for relatively low-impacted source waters, including raw drinking-water sources from major Chinese river basins (0.8–10.2 ng L^−1^) and surface/drinking waters in the Brazil (Sodré, 2018; Sun et al., 2015). In contrast, more urbanized rivers often show greater variability and higher maxima; for example, DEET in the Seyhan River system (Türkiye) reached several hundred ng L^−1^, reflecting stronger and more continuous anthropogenic pressure (Güzel, 2021). Groundwater surveys similarly indicate predominantly low‑ng L^−1^ occurrence but occasionally elevated concentrations where urban recharge or wastewater-related inputs are pronounced (Loos et al., 2010; Sorensen et al., 2015). At the source end of the continuum, substantially higher (µg L^−1^) levels are largely confined to wastewater-associated matrices such as greywater/laundry effluents and WWTP influent, consistent with direct household-use pathways (Motúzová et al., 2025). Importantly, frequent detection of DEET in runoff and in basins without clear wastewater sources indicates limited specificity as a sewage tracer; therefore, DEET is best treated as a general marker of anthropogenic activity and is most informative when evaluated alongside complementary indicators such as caffeine (Tran et al., 2014). In the present dataset, the relatively narrow concentration span and the absence of pronounced site-specific spikes suggest predominantly diffuse, activity-driven inputs rather than strong point-source sewage discharge.

The occurrence of ephedrine at trace levels (3.793–9.223 ng L^−1^) indicates that the thermal discharges are not fully isolated from the surrounding catchment and is most consistent with diluted anthropogenic inputs delivered via shallow recharge and/or near-surface mixing, rather than with in-reservoir processes within the deep geothermal system. The narrow concentration span across outlets, together with the absence of a systematic relationship with depth or temperature, further suggests that external inputs and mixing/dilution dominate variability. For context, ephedrine is commonly observed at comparable orders of magnitude in surface waters under diffuse urban pressure (Valcárcel et al., 2012) and has been reported as one of the more frequently detected stimulants across multiple Spanish basins, typically at low ng L^−1^ levels with higher values in tributaries influenced by WWTP effluents (Mastroianni et al., 2016). Municipal wastewater represents a higher-concentration end-member: in a pilot study, ephedrines were among the most abundant targets in WWTP influent (725.8 ± 181.2 ng L^−1^) while effluent concentrations were substantially lower (22.9 ± 4.9 ng L^−1^), consistent with attenuation during treatment and subsequent dilution in receiving waters (Wang et al., 2021). Field observations also indicate that increases in receiving waters can be measurable but remain modest over short distances (Rodayan et al., 2016), supporting the interpretation that the Kütahya levels reflect low-intensity, diluted inputs rather than direct sewage discharge. In groundwater, detections are generally less frequent; for example, pseudoephedrine + ephedrine was reported in recharge surface waters (2.47–6.78 ng L^−1^) but not above detection limits in sampled wells in a karst-aquifer survey (Opsahl & Musgrove, 2023), consistent with attenuation during subsurface transport and the use of ephedrine as an indicator of relatively short travel times. Finally, while screening-level prioritization approaches are useful for contextualizing potential hazards, they do not replace site-specific risk assessment (Sanderson et al., 2004); nevertheless, persistent detectability supports routine PPCPs surveillance in geothermal spa systems where shallow–deep hydraulic interaction cannot be excluded.

Chlorphenamine concentrations (0.05–0.127 ng L^−1^) showed no systematic association with discharge temperature or outlet depth, which argues against in‑reservoir generation and instead suggests a highly diluted anthropogenic imprint superimposed on the thermal matrix. This interpretation is consistent with evidence that chlorphenamine and related antihistamines may persist through conventional municipal wastewater treatment and remain detectable beyond discharge points (Kristofco & Brooks, 2017; Mar-Ortiz et al., 2020). Because PPCP datasets for thermal waters remain scarce, comparison with other aquatic compartments provides the most robust context: the sub‑ng L^−1^ levels measured here are approximately one to two orders of magnitude lower than the global median reported for rivers (6.4 ng L^−1^ across six continents) and are also below concentrations reported for reaches influenced by municipal effluents (Lissemore et al., 2006; Wilkinson et al., 2022). In this sense, the Kütahya values fall within the low end of environmental occurrence, where regional surveys commonly report intermittent detection and typically low ng L^−1^ concentrations rather than pronounced spikes (Kristofco & Brooks, 2017; Mar-Ortiz et al., 2020). The very low concentrations also emphasize an analytical consideration: some groundwater monitoring programs employ reporting limits in the several‑ng L^−1^ range (e.g., 4.68 ng L^−1^ in Minnesota’s groundwater dataset), which would not capture the sub‑ng L^−1^ signals observed here (Kroening & Vaughan, 2019). Reported removal efficiencies for pharmaceuticals in conventional treatment can be modest, enabling residual loads to persist and potentially reach shallow groundwater compartments that may locally interact with geothermal discharges via leakage, recharge, or near‑surface mixing (Aus der Beek et al., 2016; Verlicchi et al., 2012). Conversely, substantially higher concentrations reported for hospital-related wastewaters (0.293 mg L^−1^ in raw hospital wastewater) (Al-Maqrashia et al., 2024; Mar-Ortiz et al., 2020), underscore that chlorphenamine can be associated with healthcare/urban sources under direct discharge conditions. However, such a signature is not supported here given the sub‑ng L^−1^ levels and the limited detection frequency across the sampled thermal-water outlets in Kütahya. Overall, chlorphenamine in these thermal waters appears to represent a weak but measurable anthropogenic imprint, consistent with diffuse inputs and localized near‑surface mixing rather than reflecting the deep geothermal reservoir composition.

No statistically significant correlations were observed between PPCP occurrence/concentrations and the measured physicochemical properties of the thermal waters. Although the sampled waters differed in their chemical properties and well depths, the present dataset suggests that PPCP variability is not primarily controlled by these parameters. Similar observations were reported for thermal bath waters by Jakab et al. (2020). However, the absence of correlation does not by itself demonstrate source-independence or a lack of interaction; it may also reflect limited statistical power and/or the dominance of external inputs and mixing processes (Jakab et al., 2020). Furthermore, no statistically significant correlations were identified among depth (Richards et al., 2021), flow rate, temperature, and total PPCPs concentrations.

Beyond classical inorganic indicators, using caffeine and carbamazepine as a complementary tracer pair provides a more nuanced interpretation of both the timing and spatial footprint of anthropogenic inputs than relying on a single marker. Caffeine typically attenuates more readily during subsurface transport (e.g., biodegradation) and therefore can preferentially indicate recent or short‑travel‑time contributions, whereas Carbamazepine behaves more conservatively and is better suited to delineating contamination boundaries and longer‑term persistence. Accordingly, the ubiquitous detection of caffeine at ng L^−1^ levels coupled with the very low and sporadic occurrence of Carbamazepine in the present dataset is consistent with strong dilution and/or short‑travel‑time contributions from shallow, intermittently impacted flow paths. In thermal settings, such marker‑based interpretation is particularly useful because major‑ion chemistry and temperature mainly reflect deep‑water residence, whereas PPCPs can be governed by near‑surface mixing along faults or at discharge zones (Buerge et al., 2003; Dvory et al., 2018; Lukač Reberski et al., 2022; Warner et al., 2019).

The occurrence of pharmaceutical compounds in thermal water has been found to correlate strongly with the presence of modern recharge and the co-occurrence of other anthropogenic contaminants. Groundwater samples exhibiting tritium activity greater than 0.2 TU were indicative of modern water (Clark & Fritz, 1997; Lindsey et al., 2019). Most wells sampled had long screened intervals, meaning the collected groundwater represented a mixture of water of different ages.

### Potential pathways of PPCPs in Kütahya thermal waters

Overall, the PPCP signature of the Kütahya thermal water contrasts with wastewater-impacted shallow groundwater and urban surface waters where more diverse mixtures and higher concentrations are typically reported (Loos et al., 2010; Ma et al., 2022; Richards et al., 2021; Sorensen et al., 2015). This contrast supports the interpretation that PPCPs reach these geothermal outlets mainly through localized near-surface mixing and short travel-time recharge pathways rather than reflecting the deep geothermal reservoir composition.

The thermal waters of Kütahya have a long-standing history of use for therapeutic and bathing purposes, dating back to the Roman, Seljuk, and Ottoman periods. These traditional practices continue today, with many thermal areas also serving as popular sites for recreational activities such as promenades and fairgrounds especially in the summer seasons.

The location of most thermal sites in remote areas, away from urban infrastructure, has posed challenges for properly managing waste generated by thermal facilities and visitors. As a result, used thermal water is often discharged directly into the environment without adequate treatment or connection to a sewage system. This practice raises environmental concerns, as the discharge of untreated thermal water can lead to the contamination of soil and groundwater, as well as thermal waters drawn from natural springs or shallow wells.

In recent years, the integration of cosmetic applications driven by growing interest in wellness and self-care has become increasingly prevalent in thermal facilities across the region. This trend has contributed to a higher contamination load of PPCPs, microplastics, and other emerging pollutants in thermal wastewater. A study by Şener et al. (2025) detected microplastics in the thermal waters of the region at relatively low concentrations, attributing their presence to multiple potential sources, including agricultural runoff, anthropogenic activities, and improper waste disposal in and around thermal facilities (Şener et al., 2025). In this context, the presence of PPCPs in the thermal waters of Kütahya is considered to be primarily governed by two major factors: geological-hydrogeological conditions and anthropogenic inputs.

The Simav, Kütahya-Yoncalı, and Tavşanlı geothermal fields are developed along the major edge faults bordering the grabens. These grabens, characterized by intensive agricultural utilization, are infilled with young Quaternary-aged sediments, within which unconfined aquifer systems have developed at the uppermost levels. Thermal waters originating from deep geological sources ascend along fault zones and mix with shallow groundwater in an unconfined aquifer that is contaminated with anthropogenic constituents such as PPCPs and microplastics. These mixed and often contaminated thermal waters are subsequently extracted from shallow wells for use. Another significant geologic-hydrogeologic factor is the mixing of surface water contaminated with PPCPs with ascending thermal waters at discharge zones located along V-shaped valley floors, where active surface water flow is present. The Emet-Dereli, Kütahya-Ilıca, and Gediz-Abide sites are located in similar geomorphological structures, and the presence of PPCPs can be attributed to the contribution of contaminated surface waters.

The most significant anthropogenic factor, particularly affecting thermal wells and springs located in urban areas (e.g., Emet-Kaynarca), is presumed to be inadequate infrastructure or sewage system leakage, which allows PPCPs to infiltrate the soil zone and subsequently mix with the shallow groundwater that contributes to the recharge of thermal waters. Although infrastructural deficiencies in urban areas have largely been addressed, the historical pollution is still considered to continue to exert an influence on the current PPCPs load observed in thermal waters.

The primary process controlling the presence of PPCPs in thermal waters appears to be the mixing of contaminated fresh-surface water or groundwater with thermal waters in near-surface aquifers. To protect thermal waters from MPs and other contaminants, production wells should be designed to prevent any potential mixing with groundwater or surface water exposed to atmospheric conditions. To achieve this, the water intake structures/filter levels of production wells should be located in the deep reservoir zone, ensuring that they are located below the unconfined aquifer, which is at risk of contamination.

Beyond chemical analysis and quantitative risk assessment, these findings underscore the need for integrated environmental management strategies to address PPCPs in thermal water resources. The detection of pharmaceutical residues in deep thermal aquifers, which are generally considered pristine, signals a potential vulnerability in groundwater protection policies. Current regulations, both at national and EU levels, lack specific standards or monitoring requirements for PPCPs in thermal systems. Therefore, it is essential to incorporate emerging contaminants into national groundwater quality frameworks and revise existing monitoring protocols accordingly. Risk-based management strategies and early-warning monitoring systems should be developed, particularly in regions with increasing thermal tourism and therapeutic use. Furthermore, public health agencies and water resource managers must collaborate to ensure long-term sustainability and safe utilization of these natural resources.

## Conclusions

This study provides a regional assessment of pharmaceuticals and personal care products (PPCPs) in thermal‑water discharges from geothermal fields in Kütahya, Türkiye. Using a validated LC–MS/MS method, 104 target PPCPs were screened, and five compounds (caffeine, DEET, ephedrine, carbamazepine, and chlorphenamine) were quantified at low‑ng L^−1^ levels, indicating a comparatively low‑diversity PPCP signature in the investigated thermal waters. Despite the generally remote setting of many outlets, measurable PPCP concentrations show that thermal waters can receive diffuse anthropogenic inputs.

The ubiquitous detection of caffeine and the frequent occurrence of DEET, together with sporadic detections of ephedrine and chlorphenamine, are consistent with low‑intensity external inputs reaching some discharge zones, most plausibly via near‑surface pathways (e.g., shallow recharge/mixing and activity‑related releases in thermal‑use areas). Carbamazepine occurred only in a subset of samples but remains a useful conservative tracer for more persistent anthropogenic signals when interpreted alongside readily attenuated indicators such as caffeine.

Integrating the chemical results with the regional hydrogeological context suggests that fault‑controlled discharge settings can locally promote mixing between deep thermal fluids and shallow waters hosted by vulnerable unconfined aquifers and, in some cases, surface‑influenced waters. These findings support the need for integrated protection and monitoring strategies for geothermal spa systems, particularly where thermal waters are used and discharged in open, once‑through configurations. Where feasible, preventive measures such as isolating production well intakes from shallow aquifers and improving collection/treatment of spent thermal waters may reduce environmental loading and support precautionary management of thermal resources.

Overall, this regional dataset establishes a baseline for tracking emerging contaminants in geothermal discharge systems and can inform sustainable groundwater management in Türkiye and comparable hydrothermal regions.

## Supplementary Information

Below is the link to the electronic supplementary material.

Supplementary file1 (DOCX 46 KB)

## Data Availability

Data that support the findings of this study are available from the corresponding author upon reasonable request.
