# Supplementary material for: Risk assessment of a mixture of pharmaceuticals and personal care products (PPCPs) in thermal waters of Kütahya, Türkiye
Source: Environ Geochem Health. 2026 Feb 21;48(4):185. doi: 10.1007/s10653-026-03082-2 (PMC12923453; doi:10.1007/s10653-026-03082-2)
Supplement: Supplementary file 1 — Supplementary file1 (DOCX 46 KB) [file 10653_2026_3082_MOESM1_ESM.docx]

**Table S1** Sampling locations and field parameters (UTM coordinates: ED50 Zone 35).

| **No** | **District** | **Region** | **Sample Name** | **Well Depth (m)** | **Temperature (°C)** | **Flow Rate (L/s)** | **UTM X** | **UTM Y** | **Production Type** |
| --- | --- | --- | --- | --- | --- | --- | --- | --- | --- |
| 1 | Kütahya (central district) | 1 | Ilıca (Mağara) | 0 | 42 | 20 | 4387117 | 247298 | Natural spring |
| 2 | Kütahya (central district) | 2 | Yoncalı (YON-3) | 300 | 41 | 25 | 4375539 | 743805 | Pump |
| 3 |  |  | Yoncalı (YON-7) | 100 | 42 | 18 | 4375385 | 744280 | Pump |
| 4 |  |  | Yoncalı (İYJ-2) | 96 | 36.1 | 73 | 4375602 | 743396 | Pump |
| 5 |  |  | Yoncalı (İYJ-4) | 215 | 43.7 | 33 | 4376455 | 743241 | Pump |
| 6 |  |  | Yoncalı (YON-5/1) | 500 | 42.1 | 14 | 4375061 | 744244 | Pump |
| 7 |  |  | Yoncalı (İYJ-7) | 78 | 41 | 5 | 4376080 | 742987 | Pump |
| 8 |  |  | Yoncalı (Yon-1) | 301 | 43 | 8 | 4375455 | 744143 | Pump |
| 9 |  |  | Yoncalı (Mercan) | 72 | 42.8 | 5 | 4375712 | 744190 | Pump |
| 10 | Tavşanlı | 3 | Göbel | 0 | 33 | 50 | 4375027 | 709692 | Natural spring |
| 11 | Emet | 4 | Dereli Kaynak | 0 | 38.5 | 37 | 4370700 | 695069 | Natural spring |
| 12 |  |  | Dereli Şelale | 0 | 41.6 | 46 | 4370770 | 694935 | Natural spring |
| 13 | Emet | 5 | Kaynarca | 0 | 47 | 10 | 4357403 | 694527 | Natural spring |
| 14 |  |  | Yeşil Kaynağı | 0 | 45 | 11 | 4357360 | 694265 | Natural spring |
| 15 |  |  | Kükürtlü | 0 | 45 | 11 | 4357361 | 694262 | Natural spring |
| 16 | Hisarcık | 6 | Esire | 0 | 51 | 12 | 4341768 | 696527 | Natural spring |
| 17 | Simav | 7 | Eynal (EJ-4) | 602 | 152 | 30.3 | 4332727 | 671918 | Artesian |
| 18 |  |  | Eynal (E-13) | 241 | 117 | 45 | 4332825 | 672864 | Artesian |
| 19 |  |  | Çitgöl (Ç-1) | 101 | 124 | 7.1 | 4333501 | 670032 | Artesian |
| 20 |  |  | Naşa (Kuyu-1) | 65 | 85 | 1.1 | 4334557 | 669472 | Artesian |
| 21 | Gediz | 8 | Ilıca (GI-1) | 6 | 76.1 | 15 | 4312706 | 695645 | Artesian |
| 22 |  | 9 | Murat Dağı | 0 | 40.6 | 5 | 4315317 | 727210 | Natural spring |

**Table S2** List of analytes and their retention time (RT), precursor and fragment ions (m/z) for LC/MS/MS analysis

| **No** | **Analyte** | **RT (min.)** | **Precursor ion (m/z)** | **Fragment ion 1 (m/z)** | **Fragment ion 2 (m/z)** |
| --- | --- | --- | --- | --- | --- |
| 1 | 7 aminoclonezepam | 7.113 | 286.1 | 121.05 | 222.15 |
| 2 | Acetylsalicylic acid | 4.626 | 179.2 | 93.00 | 137.00 |
| 3 | Alprazolam | 7.034 | 308.9 | 281.00 | 205.10 |
| 4 | Amisulpride | 9.296 | 370.2 | 242.10 | 112.10 |
| 5 | Amitriptyline | 14.551 | 278.2 | 91.00 | 117.10 |
| 6 | Amlodipine | 15.533 | 409.2 | 238.05 | 294.10 |
| 7 | Atenolol | 9.454 | 267.2 | 190.10 | 145.10 |
| 8 | Atropine | 8.472 | 290.2 | 124.15 | 93.10 |
| 9 | Biperiden | 14.531 | 312.2 | 98.00 | 70.00 |
| 10 | Bromazepam | 8.761 | 316 | 182.20 | 209.20 |
| 11 | Buprenorphine | 10.806 | 468.3 | 396.20 | 55.10 |
| 12 | Caffeine | 4.428 | 195.1 | 138.15 | 42.15 |
| 13 | Carbamazepine | 6.923 | 237.1 | 193.10 | 194.10 |
| 14 | Chlordiazepoxide | 7.951 | 300.1 | 227.20 | 282.20 |
| 15 | Chlorpheniramine | 12.722 | 275.1 | 167.20 | 230.20 |
| 16 | Chlorpromazine | 14.496 | 319.1 | 86.10 | 58.00 |
| 17 | Citalopram | 13.653 | 325.2 | 109.10 | 262.20 |
| 18 | Clobazam | 9.657 | 301.1 | 224.20 | 259.20 |
| 19 | Clomipramine | 15.842 | 315.2 | 86.10 | 58.00 |
| 20 | Clonazepam | 8.424 | 316 | 241.20 | 270.20 |
| 21 | Clozapine | 10.366 | 327.1 | 192.20 | 270.10 |
| 22 | Codein | 6.628 | 300.2 | 165.20 | 153.20 |
| 23 | DEET | 7.811 | 192.2 | 91.10 | 119.10 |
| 24 | Desipramine | 9.263 | 267.2 | 72.10 | 193.20 |
| 25 | Desmethylclomipramine | 12.483 | 301.2 | 72.15 | 228.05 |
| 26 | Dextrometorphan | 15.594 | 272.2 | 171.20 | 147.20 |
| 27 | Diazepam | 8.492 | 285.1 | 154.20 | 222.30 |
| 28 | Diazepam-d5 (IS) | 8.509 | 290 | 227.20 | 198.20 |
| 29 | Diclofenac | 7.494 | 296.1 | 215.05 | 214.10 |
| 30 | Dihydroergotamine | 9.935 | 584.2 | 270.15 | 253.20 |
| 31 | Diltiazem | 6.57 | 415.2 | 178.05 | 150.05 |
| 32 | Diphenhydramine | 12.35 | 256.1 | 167.05 | 152.10 |
| 33 | Doxepine | 12.614 | 279.8 | 107.00 | 235.00 |
| 34 | Doxylamine | 13.036 | 271.2 | 167.10 | 182.20 |
| 35 | ephedrine | 7.219 | 166.1 | 148.15 | 117.10 |
| 36 | Erythromycin | 15.599 | 734.3 | 158.10 | 576.30 |
| 37 | Escitalopram | 13.68 | 325.2 | 109.10 | 262.10 |
| 38 | Etodolac | 7.749 | 288.2 | 172.15 | 143.15 |
| 39 | Famotidine | 10.819 | 338.1 | 135.10 | 189.10 |
| 40 | Fentanyl | 11.133 | 337.3 | 188.15 | 105.05 |
| 41 | Fluconazole | 5.608 | 307.1 | 220.15 | 238.10 |
| 42 | Flunitrazepam | 8.147 | 314.1 | 239.20 | 268.20 |
| 43 | Fluoxetine | 12.057 | 310.1 | 117.10 | 91.10 |
| 44 | Flurazepam | 10.925 | 388.2 | 315.10 | 317.10 |
| 45 | Fluvoxamine | 9.746 | 319.2 | 200.10 | 71.00 |
| 46 | Gabapentin | 4.929 | 172.15 | 154.10 | 55.10 |
| 47 | Haloperidol | 12.726 | 376.1 | 123.00 | 165.10 |
| 48 | Hydroxyzine | 11.241 | 375.2 | 166.10 | 201.10 |
| 49 | Ibuprofen | 7.752 | 205.1 | 161.20 | 89.10 |
| 50 | Imipramine | 14.888 | 281.2 | 86.10 | 58.00 |
| 51 | Ketamine | 8.962 | 238.1 | 125.00 | 179.10 |
| 52 | Lidocaine | 9.377 | 235.2 | 86.00 | 58.00 |
| 53 | Loperamide | 13.856 | 477.3 | 266.10 | 210.20 |
| 54 | Lorazepam | 10.738 | 321 | 275.10 | 303.10 |
| 55 | Medazepam | 8.427 | 270.9 | 91.05 | 207.15 |
| 56 | Mescaline | 7.293 | 212.1 | 91.10 | 77.10 |
| 57 | Metformin | 2.091 | 130.1 | 60.10 | 71.10 |
| 58 | Methadone | 12.108 | 309.7 | 265.15 | 105.20 |
| 59 | Metoclopramide | 9.944 | 300.2 | 227.00 | 184.00 |
| 60 | Metoprolol | 10.557 | 268.2 | 56.1 | 77.00 |
| 61 | Mianserin | 10.453 | 265.2 | 208.20 |  |
| 62 | Midazolam | 10.691 | 325.9 | 291.05 | 221.95 |
| 63 | Mirtazepine | 9.552 | 266.2 | 195.20 | 194.20 |
| 64 | Moclobemide | 7.508 | 269.1 | 139.10 | 182.10 |
| 65 | Morfin | 4.758 | 286.1 | 165.10 | 153.20 |
| 66 | Naproxen | 7.755 | 229.1 | 56.30 | 106.10 |
| 67 | Nifedipine | 8.881 | 347.2 | 315.00 | 328.95 |
| 68 | Nitrazepam | 12.652 | 282.1 | 180.20 | 236.20 |
| 69 | Nordiazepam | 13.06 | 271.1 | 165.10 | 91.10 |
| 70 | Nortriptilin | 9.579 | 264.2 | 91.00 | 117.10 |
| 71 | Olanzapine | 10.411 | 313.1 | 198.10 | 256.20 |
| 72 | Opipramol | 11.486 | 364.2 | 143.20 | 171.20 |
| 73 | Ornidazole | 13.986 | 220.2 | 105.20 | 127.90 |
| 74 | Oxazepam | 10.993 | 287.1 | 269.20 | 241.20 |
| 75 | Oxcarbazapine | 6.04 | 253.1 | 180.10 | 208.10 |
| 76 | Pantoprazole | 16.01 | 382.1 | 135.10 | 107.10 |
| 77 | Paracetamol | 1.835 | 152.2 | 110.10 | 65.10 |
| 78 | Paroxetine | 9.41 | 330.1 | 69.90 | 192.20 |
| 79 | Pentobarbital | 12.043 | 225.1 | 101.70 | 110.95 |
| 80 | Pentoxifylline | 11.846 | 279.2 | 58.05 | 181.10 |
| 81 | Pethidine | 11.416 | 248 | 174.10 | 220.10 |
| 82 | Pheniramine | 11.038 | 241.2 | 167.20 | 196.20 |
| 83 | Phenobarbital | 9.356 | 231 | 115.05 | 70.95 |
| 84 | Phenytoin | 13.062 | 253.1 | 180.10 | 236.05 |
| 85 | pregabalin | 3.554 | 160.1 | 142.10 | 55.10 |
| 86 | Prilocaine | 6.915 | 221.2 | 86.10 | 136.10 |
| 87 | Propafenon | 9.438 | 342.2 | 116.10 | 72.15 |
| 88 | Propranolol | 16.132 | 260.2 | 116.10 | 56.00 |
| 89 | Propyphenazone | 7.538 | 231.2 | 189.15 |  |
| 90 | Pseudoephedrine | 7.287 | 166.1 | 91.00 | 115.00 |
| 91 | Quetiapine | 9.52 | 384.2 | 253.10 | 221.15 |
| 92 | Risperidone | 8.516 | 411.2 | 191.10 | 148.20 |
| 93 | Sertraline | 10.474 | 306.1 | 159.00 | 275.10 |
| 94 | Sildenafil | 9.489 | 475.2 | 58.10 | 100.10 |
| 95 | Sulfamethoxazole | 9.189 | 253.9 | 92.10 | 156.00 |
| 96 | Sulphadiazine | 13.062 | 251 | 92.10 | 156.00 |
| 97 | Thiopental | 10.958 | 241.2 | 51.10 | 178.50 |
| 98 | Thioridazine | 10.831 | 371.2 | 98.10 | 126.10 |
| 99 | Tramadol | 9.468 | 264.2 | 56.1 | 58.1 |
| 100 | Triamethoprim | 8.512 | 291 | 230.10 | 261.10 |
| 101 | Triclosan | 5.042 | 288.7 | 35.00 | 36.95 |
| 102 | Triclosan-d3 (IS) | 5.057 | 289.8 | 34.95 |  |
| 103 | Trimipramine | 9.446 | 295.2 | 100.15 | 58.15 |
| 104 | Venlafaxine | 11.66 | 278.2 | 121.10 | 58.00 |
| 105 | Verapamil | 12.245 | 455.3 | 165.10 | 150.10 |
| 106 | Yohimbine | 9.996 | 355.1 | 144.10 | 212.10 |

**Table S3** Toxicity data of detected PPCPs in thermal water for algae, ivertebrates and fish.

| Compound Name | CAS No | Test organisms | EC50/LC50  (mg/L)* | Duration | AF | PNEC  (ng/L) |
| --- | --- | --- | --- | --- | --- | --- |
| Caffeine | 58-08-2 | Green algae (estimated) | 0.015 | 96 h | 1000 | 15 |
|  |  | Daphnid | 92.6 | 48 h |  |  |
|  |  | Fish | 329 | 96 h |  |  |
| DEET | 134-62-3 | Green algae (estimated) | 4.48 | 96 h | 1000 | 4480 |
|  |  | Daphnid | 31.4 | 48 h |  |  |
|  |  | Fish | 33.6 | 96 h |  |  |
| Ephedrine | 299-42-3 | Green algae (estimated) | 26.6 | 96 h | 1000 | 26600 |
|  |  | Daphnid | 23.8 | 48 h |  |  |
|  |  | Fish | 233 | 96h |  |  |
| Carbamazepine | 298-46-4 | Green algae (estimated) | 0.260 | 96 h | 1000 | 260 |
|  |  | Daphnid | 14.1 | 48 h |  |  |
|  |  | Fish | 40.9 | 96 h |  |  |
| Chlorphenamine | 132-22-9 | Green algae  (estimated) | 0.269 | 96 h | 1000 | 269 |
|  |  | Daphnid | 0.496 | 48 h |  |  |
|  |  | Fish | 3.37 | 96 h |  |  |

*EC50 and LC50 values were obtained using Ecological Structure Activity Relationships (ECOSAR) Predictive Model v2.2 software programme.
